# Implementing evidence into practice to improve chronic lung disease management in Indigenous Australians: the breathe easy, walk easy, lungs for life (BE WELL) project (protocol)

**DOI:** 10.1186/s12890-022-02033-8

**Published:** 2022-06-21

**Authors:** David P. Meharg, Christine R. Jenkins, Graeme P. Maguire, Stephan Jan, Tim Shaw, Sarah M. Dennis, Zoe McKeough, Vanessa Lee, Kylie G. Gwynne, Debbie McCowen, Boe Rambaldini, Jennifer A. Alison

**Affiliations:** 1 Faculty of Medicine and Health, Sydney School of Health Sciences, The University of Sydney, Camperdown, NSW 2006 Australia; 2 Poche Centre for Indigenous Health, The University of Sydney, Camperdown, NSW 2006 Australia; 3 The George Institute for Global Health, Newtown, NSW 2042 Australia; 4 Faculty of Medicine, University of New South Wales, Sydney, Kensington, NSW 2052 Australia; 5 Curtin Medical School, Faculty of Health Sciences, Curtin University, Bentley, WA 6102 Australia; 6 South Western Sydney Local Health District, Liverpool, NSW 2170 Australia; 7 Ingham Institute for Applied Medical Research, Liverpool, NSW 2170 Australia; 8 Faculty of Medicine, Health and Human Sciences, Macquarie University, North Ryde, NSW 2109 Australia; 9 Armajun Aboriginal Health Service, Inverell, NSW 2360 Australia; 10 Sydney Local Health District, Camperdown, NSW 2050 Australia

**Keywords:** Indigenous, Chronic obstructive pulmonary disease (COPD), Pulmonary rehabilitation, Aboriginal community controlled health service, Implementation science

## Abstract

**Background:**

Strong evidence exists for the benefits of pulmonary rehabilitation (PR) for people with chronic obstructive pulmonary disease (COPD), however the availability of culturally safe PR for Aboriginal and Torres Strait Islander (Indigenous) Peoples is limited. The study aims to determine whether PR can be implemented within Aboriginal Community Controlled Health Services (ACCHS) to improve outcomes for Indigenous people with COPD.

**Methods:**

Multi-centre cohort study using participatory action research guided by the Knowledge-to-Action Framework. ACCHS supportive of enhancing services for chronic lung disease will be recruited. Aboriginal Health Workers (AHW) and the exercise physiologist (EP) or physiotherapist (PT) within these ACCHS will attend a workshop aimed at increasing knowledge and skills related to management of COPD and the provision of PR. Indigenous people with COPD will be invited to attend an 8-week, twice weekly, supervised PR program. Outcomes: AHW, EP/PT knowledge, skills and confidence in the assessment and management of COPD will be measured before and immediately after the *BE WELL* workshop and at 3, 6 and 12 months using a survey. PR participant measures will be exercise capacity (6-minute walk test (6MWT), health-related quality of life and health status at commencement and completion of an 8-week PR program. Secondary outcomes will include: number, length and cost of hospitalisations for a COPD exacerbation in 12-months prior and 12-months post PR; local contextual factors influencing implementation of PR; specific respiratory services provided by ACCHS to manage COPD prior to project commencement and at project completion. Repeated measures ANOVA will be used to evaluate changes in knowledge and confidence over time of AHWs and EP/PTs. Paired t-tests will be used to evaluate change in patient outcomes from pre- to post-PR. Number of hospital admissions in the 12 months before and after the PR will be compared using unpaired t-tests.

**Discussion:**

Pulmonary rehabilitation is an essential component of best-practice management of COPD and is recommended in COPD guidelines. Indigenous peoples have limited access to culturally safe PR programs. This study will evaluate whether PR can be implemented within ACCHS and improve outcomes for Indigenous people with COPD.

*Trial registration* Australian New Zealand Clinical Trials Registry (ANZCTR) ACTRN12617001337369, Registered 2nd September 2017 https://www.anzctr.org.au/Trial/Registration/TrialReview.aspx?id=373585&isClinicalTrial=False

## Background

Chronic obstructive pulmonary disease (COPD) affects 1.5 million Australians, including 1 in 13 people over 40 years of age [1] with major consequences for participation in work and societal contexts. Aboriginal and Torres Strait Islander Peoples of Australia, hereafter referred to as Indigenous Australians, bear an unequal burden of disease in relation to COPD. Compared to non-Indigenous Australians, the prevalence of COPD is 2.5 times higher, with the death rate being three times higher and the hospitalisation rate five times higher [2]. Importantly, COPD is the greatest contributor to potentially preventable hospitalisations in Indigenous Australians, [3–5] which are those hospitalisations that may have been prevented by provision of evidenced-based interventions delivered in primary care [4].

Pulmonary rehabilitation (PR), a program of exercise training and education, is recommended as best-practice care for people with COPD [6] underpinned by Level 1 evidence from over 85 randomised controlled trials in two Cochrane reviews [7, 8]. These reviews concluded that pulmonary rehabilitation improves exercise capacity, reduces symptoms of breathlessness and fatigue, improves health-related quality of life, and reduces hospital admissions and mortality in people with COPD [7, 8]. Reducing hospitalisations is not only important for reducing health care costs, but a valuable outcome for Indigenous people since hospitalisations cause dislocation from family, especially if hospitals are distant from the communities in which they live. Reducing mortality from COPD is also vital in addressing the gap in life expectancy experienced by Indigenous compared to non-Indigenous Australians [2].

In Indigenous Australian communities there have been coordinated approaches to improve the management of other chronic diseases such as diabetes, cardiovascular and renal disease, however efforts to improve the management of chronic lung diseases, such as COPD, have been inadequate and inconsistent. A major reason for limited access to best-practice management of COPD is the perception by Indigenous peoples that PR programs offered in centre-based hospital outpatient departments are unwelcoming or culturally unsafe [9] due to racism [10]. Additionally there is a lack of knowledge, skills and confidence of healthcare professionals, as well as lack of funding, to implement PR programs in rural and remote Australia [11], including within Aboriginal Community Controlled Health Services (ACCHS). This means that Indigenous peoples with COPD are much less likely to receive best-practice management for COPD and more likely to have poorer health outcomes.

A recent systematic review of PR for Indigenous peoples with COPD in Australia, Canada, New Zealand and the United States of America found only one published study reporting the outcomes of PR, [12] highlighting the paucity of data evaluating PR in Indigenous communities.

## Aim and objectives

The broad aim of the *Breathe Easy Walk Easy, Lungs for Life (BE WELL)* project is to evaluate the implementation of lung health services within ACCHS, particularly the provision of PR.

The implementation objectives (1–3 in the list below) and the intervention objective (4 in the list below) of the study are to:

1. Evaluate the ability of the *BE WELL* program to enhance the knowledge, skills and confidence of Aboriginal Health Workers in the assessment and management of people with COPD and in the delivery of a culturally safe PR program that includes exercise training and patient education.
2. Identify the structural, systems and other contextual factors that influence successful implementation and sustainability of PR programs within ACCHS.
3. Determine the uptake of respiratory assessment and PR programs by ACCHS
4. Determine the impact of the *BE WELL* PR program on health outcomes and health care utilisation and costs (particularly hospitalisations) of Indigenous people with COPD.

## Methods/design

**Fig. 1:**
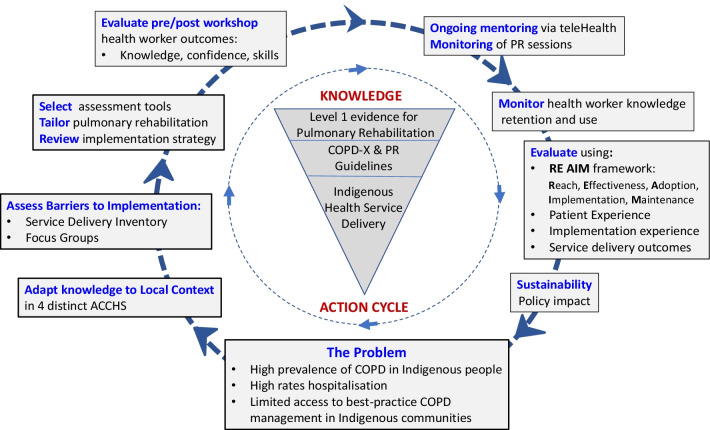
Knowledge-to-Action Framework. *ACCHS* Aboriginal community controlled health service; *COPD* Chronic obstructive pulmonary disease; *COPD-X* Australian COPD guidelines; *PR* Pulmonary rehabilitation

The study will be a cohort study using implementation science and participatory action research methods guided by the Knowledge-to-Action Framework [13–15]. This will be an iterative process of reflection and action carried out with and by ACCHS rather than ‘on’ them [15]. Fig. 1 maps the knowledge already established for PR and the elements of the *BE WELL* program in the Knowledge-to-Action Framework. The study has ethics approval from the Aboriginal Health and Medical Research Council of New South Wales, Australia (Project 1261/17). All participants will provide written, informed consent. Any protocol amendments will be approved by the ethics committee and reported on the Australian New Zealand Clinical Trials Registry.

## Participants

ACCHS in New South Wales, Australia will be invited to participate in the study with the aim of recruiting one ACCHS from each of the following regions: metropolitan, regional, rural, remote. These regions will be identified by the remoteness classification [16]. Initial engagement of ACCHS will be facilitated by the Poche Centre for Indigenous Health, University of Sydney.

Within the ACCHS the following participants will be recruited: (1) ACCHS management who have oversight or involvement in managing any aspects of the *BE WELL* project; (2) Aboriginal health workers (AHWs) who are nominated by the ACCHS to work within the local *BE WELL* team to develop and aid provision of a PR program; (3) Exercise physiologists (EP) or physiotherapists (PT) who are contracted by the ACCHS to provide the PR program within the ACCHS; (4) Aboriginal people attending the ACCHS who have a confirmed diagnosis of COPD by spirometry [ratio of forced expiratory volume in one second (FEV_1_) to forced vital capacity (FVC) of less than < 0.7] and who are referred to the *BE WELL* PR program.

The implementation within each ACCHS will consist of:

- Consultation with each of the participating ACCHS. Members of the research team [DM1 (Indigenous), JA (non-Indigenous)] will visit each ACCHS at the start of the project to discuss the *BE WELL* program in more detail. A focus group will explore the local structural, system level and other contextual factors that will influence successful implementation within the ACCHS. The discussion, guided by the Theoretical Domains Framework, [17] will enable the researchers and local stakeholders to develop a local implementation plan that addresses some of the identified barriers or enablers. Feedback from this consultation will be provided to the stakeholders and will help to inform the implementation strategy. At this visit the *BE WELL Service Delivery Inventory*, which records services currently provided by the ACCHS to manage chronic lung disease, will be completed.
- A face-to-face workshop for AHWs and the EP or PT who will be responsible for providing the PR program. The workshop will be a local two-day *BE WELL* workshop at their site. The workshop will be provided by a physiotherapist experienced in pulmonary rehabilitation and adult education (JA). The workshop will incorporate the Aboriginal pedagogy of 8 Ways of Learning [18] and will include education and practical skills in all components of comprehensive pulmonary rehabilitation, including: (a) the pathophysiology of COPD and bronchiectasis (bronchiectasis frequently coexists with COPD in Indigenous Australians); [19] (b) patient assessment of lung function by spirometry, exercise capacity health-related quality of life, health status; (c) patient exercise prescription and exercise training; (e) patient education; (f) evaluation of patient outcomes at completion of PR. Educational resources presented at the workshop will be available to each ACCHS. All information regarding PR will be evidence-based [20] and supported by the web-based Pulmonary Rehabilitation Toolkit [21].
- Online education. To enable patient education (‘yarning’) sessions to be co-designed with the AHWs and EP/PT, online education sessions will be provided. ‘Yarning’ is the process of holding informal conversations that prioritise respectful and cooperative Indigenous ways of communicating as it enables connectedness, accountability and cultural safety [22]. The online education will consist of weekly zoom meetings with the AHWs and EP/PT from each participating ACCHS. At these meetings the physiotherapist (JA) and member of the research team (DM1) will provide information about one of the education topics, guided by the 8 ways of Aboriginal learning [18]. The AHW, with the support of the EP/PT, will then develop a yarning script and implement an online role play relevant to the Aboriginal community where the yarning will be held. This process will continue until the following seven topics have been covered: How the lungs work; What is COPD; Medications and how to use inhalers and COPD Action Plans; Why exercise is important; Managing breathlessness; Healthy eating; Managing anxiety and depression.

The intervention will consist of provision of a PR program run by EP/PT and AHWs. Participants with COPD will be asked to attend twice a week for eight weeks. The PR program will consist of patient assessment, exercise prescription and training, patient education, and patient reassessment at program completion as outlined below.

- *Patient Assessment* will include medical history; lung function testing using spirometry (MIR Spirodoc Spirometer, Rome, Italy); evaluation of exercise capacity using the six-minute walk test (6MWT) performed twice and using standard procedures [23] including measurement of oxygen saturation and pulse rate each minute using a pulse oximeter (Spirodoc); evaluation of health-related quality of life using the St George’s Respiratory Questionnaire (SGRQ) [24] and the EuroQual-5D-5L questionnaire (EQ5D5L) [25]; health status using the COPD Assessment Test (CAT) [26]. Each ACCHS will be provided with equipment needed to perform patient assessment and monitoring during pulmonary rehabilitation, including a spirometer combined with a pulse oximeter (Spirodoc).
- *Exercise prescription and training* will include walking, stationary cycling and resistance training. Walking training intensity will be based on walking at 80% of the better 6MWT speed and will either be walking on a flat indoor track or on a treadmill. Cycle training intensity will be 60% of peak work rate calculated from the 6MWT [27]. Participants will start with 10 min walking and 10 min cycling which will be progressed to 20 min walking and 20 min cycling over the first 3 weeks of the program. Intensity will be progressed by increasing walking speed on the treadmill or indoor track and work rate (watts) on the cycle ergometer to keep the symptom scores of dyspnoea or perceived exertion (whichever is the highest) at 3–4 (‘moderate' to ‘somewhat severe’) on the 0–10 category-ratio scale [28]. Resistance training will use elastic resistance bands and simple hand weights for upper limbs and body weight exercises such as squats, sit-to-stand, step-ups for lower limbs. To ensure equity of equipment, each ACCHS will be provided with a treadmill, cycle ergometer and elastic resistance bands. Prior to starting the PR program, participants will be assessed by a general medical practitioner to optimise medications and to ensure that there are no contraindications to exercise training.
- *Patient education* ‘yarning’ sessions’, led by the AHW, will occur once a week at the end of one of the exercise sessions. There will be seven topics for patient education (see point 3 above).
- *Patient reassessment* at the end of the PR program will use the 6MWT, SGRQ, CAT and EQ5D-5L. Spirometry will also be performed to determine whether the participant’s lung function remained stable during the pulmonary rehabilitation program. Participants will also be asked to complete a survey of their experience of pulmonary rehabilitation (Table1).

**Table 1:** Study objectives and outcome measures

Objectives	Outcome measure	Purpose	Description	Participants	When administered
*Implementation*					
Objective 1: Enhance health provider knowledge, skills and confidence	*BE WELL* Health Provider Impact Questionnaire	To evaluate the effects of the *BE WELL* workshop on health care provider knowledge, confidence and skills	The questionnaire will include a Likert scale to assess self-reported knowledge of COPD, and skills in assessing people with COPD (including spirometry and 6MWT), and confidence in exercise prescription (EP/PTs only) and providing an exercise program and education for Indigenous people with COPD	AHWs and EPs/PTs who attend the *BE WELL* workshop	Before and immediately after the *BE WELL* workshop, and at 3, 6 and 12 months post-workshop
	Online education survey	To evaluate the AHW and EP/PT experience of the online education sessions for developing ‘yarning’ educational resources for *BE WELL* participants undertaking the PR program	A 15-question survey using a 5-point Likert scale evaluating the mode of delivery, structure of the sessions, and engagement	AHWs and EPs/PTs who attend the *BE WELL* online education sessions	After completion of the online education sessions at each participating ACCHS
	Online education semi-structured interviews	To explore the AHW and EP/PT experiences of the online education sessions to completement the responses from the online education survey	To guide discussion, 13 interview questions covering context, co-design, engagement, knowledge and understanding, cultural integration and impact will be used. The interviews will be conducted by a member of the research team experienced in qualitative interviews and who is not involved in the delivery of the online education sessions	AHWs and EPs/PTs who attend the *BE WELL* online education sessions	After completion of the online education sessions at each participating ACCHS
Objective 2: Identify factors that influence successful implementation	Focus groups	To explore the local structural, system level and other contextual factors that could influence successful implementation within each ACCHS	To guide discussion there will be questions relating to the elements of the RE-AIM framework i.e. Reach, Effectiveness, Adoption, Implementation, Maintenance	ACCHS staff involved in *BE WELL* project	At project inception and at 12-months after the *BE WELL* workshop
	Survey	To gain feedback from patients about the *BE WELL* PR program	Survey using 5-point Likert scale to evaluate patients’ knowledge of lung disease, confidence in self-management, program satisfaction and suggestions for improvement	Indigenous patients enrolled in the *BE WELL* PR program	At the completion of each patient’s PR program
	Focus groups	To gain a more in-depth understanding of the patient experience of the *BE WELL* PR program	To guide discussion there will be questions relating to patients’ experiences of the *BE WELL* PR program and patients’ perceived changes in knowledge of lung disease, confidence in self-management, and their degree of satisfaction with the program. Advice will be sought from each ACCHS to determine the most culturally appropriate method of engaging with patients	Indigenous patients enrolled in the *BE WELL* PR program	After completion of PR by a group of participants
Objective 3: Uptake of new services for COPD management	*BE WELL* Service Delivery Inventory	To evaluate the extent of respiratory services at each ACCHS and the impact of the *BE WELL* program on the services provided	The inventory will cover the following services: spirometry for assessment of COPD, assessment of smoking status, provision of evidence-based smoking cessation advice and/or treatment, provision of pulmonary rehabilitation programs that include exercise training and patient education	Executive staff of each ACCHS	At initial consultation and at project completion
*Intervention*					
Objective 4: Impact of *BE WELL* PR program on patient outcomes and health care costs	6MWT (23)	To evaluate functional exercise capacity	Measures distance walked in 6 min on a flat, indoor track. Two tests will be performed at baseline and one test at program completion. Oxygen saturation and pulse rate will be recorded continuously using a pulse oximeter (MIR Spirodoc Spirometer, Rome, Italy)	Indigenous patients attending the *BE WELL* PR program	Before and after participation in the 8-week *BE WELL* PR program
	SGRQ (24)	To evaluate HRQoL	A 50-item questionnaire with domains of impact, symptoms and activity impairment associated with COPD		
	EQ5D-5L (25)	To evaluate HRQoL	A generic quality of life questionnaire consisting of five dimensions (mobility, self-care, usual activities, pain/discomfort, anxiety/depression) which will be used in cost-effectiveness analysis		
	CAT (26)	To evaluate the impact that COPD on wellbeing and daily life	An 8-item questionnaire evaluating symptoms, activity limitations, sleep, confidence		
	Hospitalisations	To evaluate the impact of the *BE WELL* PR program on hospitalisations	Hospital separation data will be collected from the NSW Centre for Health Record Linkage (CHeReL). Administrative hospital records including information on the Diagnostic Related Group (DRG) classification and International Classification of Disease (ICD-10) codes for hospital separations will be used to estimate a cost using local cost weights	Indigenous patients who participated in the *BE WELL* PR program	In the periods 12 months preceding and following participation in the *BE WELL* PR program
	Other healthcare costs (GP visits, medications)	To evaluate the impact of the *BE WELL* PR program on other healthcare costs	Costs of medical services and medications will be recorded from patients who consent to access of their administrative health care use data through the Medical and Pharmaceutical Benefits Schedules (PBS) from Medicare Australia. Additional primary health care and medication use data will be abstracted from primary health care histories particularly in sites where the PBS have limited implementation, e.g. very remote Australian health care services		
	Costs of *BE WELL* pulmonary rehabilitation program	To determine the costs of provision of the *BE WELL* PR program for comparison with any costs savings	Costs of program delivery will include staff time, facility costs, training resources	Executive staff of the ACCHS	During the *BE WELL* project

*ACCHS* Aboriginal community controlled health services, *AHW* Aboriginal health worker, *BE WELL* Breathe easy walk easy lungs for life, *CAT* COPD assessment test, *COPD* Chronic obstructive pulmonary disease, *EP/PT* Exercise physiologist/physiotherapist, *EQ5D-5L* EuroQual 5 dimensions-5 levels, *GP* General practitioner, *HRQoL* Health-related quality of life, *PR* Pulmonary rehabilitation, *SGRQ* St George’s respiratory questionnaire, *6MWT* Six-minute walk test

## Outcome measures

The *BE WELL* program will be evaluated using the RE-AIM Framework of Reach, Effectiveness, Adoption, Implementation and Maintenance [29] as well as patient experience of PR and ACCHS experience of implementing PR. For RE-AIM, Reach will be assessed by the number of participants with COPD who attend the *BE WELL* program in relation to the number of people with COPD being managed by each participating ACCHS. Effectiveness will be assessed by the outcomes of participants in response to the *BE WELL* PR program, particularly improvements in exercise capacity and health-related quality of life. Adoption will be assessed by the uptake of the *BE WELL* PR program by patients with COPD and by the AHWs involved in the delivery of the program. The barriers and facilitators to adoption by people with COPD and AHWs will be reported. Implementation will be assessed by determining whether the *BE WELL* PR program was provided as intended. Maintenance will be determined by the number of ACCHS that continue to offer the *BE WELL* PR program at completion of the project. The intervention outcomes will be measured by the objective assessments of the participants before and at completion of the *BE WELL* PR program. The outcome measures specifically related to the main study objectives and the timing of these measures are detailed in Table 1.

## Reference group

A *BE WELL* Reference Group will be established at the commencement of the project. The Reference Group will consist of representatives from Aboriginal communities and organisations, and from relevant State and Federal health care and professional bodies. This group will provide expertise and guidance throughout the project, will ensure that the project initiatives fit with the organisational, policy and cultural context, and will advise on strategies to enable sustainable implementation and transferability to the broader ACCHS sector.

## Data and data analysis

Plans for data entry, coding, security, and storage will be held on the secure Research Management Dashboard of the University of Sydney. Quantitative data will be collated and analysed using SPSS statistics package. Scores from the *BE WELL Health Provider Impact Questionnaire* before and immediately after the workshop will be compared using paired t-tests. Repeated measure ANOVA will be used to evaluate knowledge retention over time. For categorical measures of self-rated knowledge, confidence and skills, change scores will be calculated and analysed using Wilcoxon’s signed ranks test. Patient outcomes for exercise capacity and health-related quality of life will be compared before and after PR using paired t-tests. The number of hospital admissions for respiratory related care in the 12 months before and after the *BE WELL* PR program will be compared using unpaired t-tests and the number of patients admitted before and after program will compared using proportions. The costs of delivery of the *BE WELL* program will be determined and potential savings against reductions in health care utilisation will be quantified [30]. For qualitative data, the focus groups with ACCHS staff and with participants in the *BE WELL* PR program and interviews with AHW who participate in the online education sessions will be digitally recorded and transcribed. Data will be analysed in QSR International NVivo using inductive coding and thematic analysis [31]. Any adverse events will be reported immediately to the ethics committee and will be reported in any publications.

## Sample size

Sample sizes have been calculated for the quantitative measurements. For evaluating changes in health worker knowledge of COPD management, the scores from the objective knowledge test in *BE WELL Health Provider Impact Questionnaire* will be used. The sample size, based on pilot data demonstrating change in knowledge score of 7 marks [standard deviation (SD) 4.5] [32], requires eight AHW participants, with a power of 80%, alpha 0.05, allowing for a 20% dropout (i.e. two AHWs per ACCHS). For the patient outcomes, based on our pilot study in rural and remote areas, to detect a change in 6MWT of 48 m, SD 70 m [11], with a power of 80% and alpha 0.05, 40 patients will be required, allowing for a 15% dropout. For quality of life, to detect a minus 4-point improvement in SGRQ (which is the minimal important difference [33]) with SD 6 points, 42 patients will be required, allowing for a 15% dropout, with power 80% and alpha 0.05. For hospitalisations, we have conservatively assumed a 20% reduction in hospital admissions. Therefore 97 patients would be needed, power 80%, alpha 0.05, allowing for 15% drop-out. Based on the largest patient sample size required, 97 patients with COPD will be recruited. For qualitative data, focus groups and interviews will be undertaken with participants until no new information is obtained and saturation is achieved.

## Discussion

This study aims to determine whether PR can be implemented in ACCHS to improve outcomes for Indigenous people with COPD. As stated in the 4th Atlas of Clinical Variation there is an unacceptably high rate of hospitalisations for people with COPD generally, and for Indigenous Australians in particular, and strategies known to improve the health of people with COPD need to be implemented [5]. This is especially important for Indigenous people who have five times the hospitalisation rate and three times the mortality for COPD than non-Indigenous people [2].

The availability of culturally safe PR programs for Indigenous people with COPD is minimal since mainstream PR services are usually provided in hospital outpatient departments which Indigenous people are unlikely to access given their distrust of the hospital system [34], highlighting the need for ACCHS to provide such services. ACCHS are primary care services [35] initiated and operated by the local Aboriginal community to deliver holistic, comprehensive, and culturally appropriate health care to their community and were established due to the inability to properly engage Indigenous peoples in mainstream Australian health services [36]. Programs of rehabilitation for chronic lung disease are not usually core business of ACCHS which is exemplified by a recent systematic review which was only able to report one study where a PR program was provided within an ACCHS [12]. This paucity of data on the implementation of PR programs within ACCHS and the uptake by Indigenous people with COPD needs to be addressed if we are to close the gap in outcomes. The *BE WELL* project will provide a better understanding of what is needed within ACCHS to enable them to provide PR programs for their community members who have COPD.

A strength of the study is the implementation science and participatory research methodology using the Knowledge-to-Action framework [14]. This study design was chosen rather than a randomised trial design since the patient intervention, PR, has been well established as effective for non-Indigenous people with COPD [7, 8] and the purpose of this project is to determine if PR can be implemented within Indigenous communities through a co-design process. The Knowledge-to-Action framework with participation from the ACCHS enables iterative processes of reflection and action carried out with and by the ACCHS rather than ‘on’ them and will ensure that local context can be incorporated into the *BE WELL* PR program so that it fits with the needs and the physical and staffing infrastructure of each ACCHS. As such, modifications to the delivery of the PR program can be made if needed, based on understanding the local barriers and facilitators to program delivery by partnering with each ACCHS. A further strength of the study is the focus on better understanding the requirements of the AHW workforce to upskill in the management of the major health issue of COPD. The *BE WELL* project will provide an understanding of the educational requirements and clinical experiences needed to upskill AHWs so that they can be actively engaged in this area of practice in ACCHS.

The findings of this study will contribute important information to the understanding of what enables an ACCHS to provide lung health services, particularly PR. Given the high burden and high cost of COPD for Indigenous people, improving quality of life and reducing hospital admissions and mortality through the delivery of PR is highly relevant to the major Indigenous health priorities to close the gap in life expectancy within a generation (by 2031) [37]. The *BE WELL* program aims to ensure equity of access for Indigenous people to effective interventions for COPD.

## Data Availability

Not applicable. The manuscript does not contain any data. Future publications of study findings will be published in per-reviewed journals with participant data de-identified for confidentiality. Authorship will be based on contribution.

## Abbreviations

ACCHS: Aboriginal community controlled health services
AHW: Aboriginal health worker
ANOVA: Analysis of variance
BE WELL: Breathe Easy Walk Easy Lungs for Life
CAT: COPD assessment test
COPD: Chronic obstructive pulmonary disease
COPD-X: Australian COPD guidelines
EP: Exercise physiologist
EQ5D5L: EuroQol 5 dimension 5 level questionnaire
FEV_1_: Forced expiratory volume in one second
FVC: Forced vital capacity
PR: Pulmonary rehabilitation
PT: Physiotherapist
RE-AIM: Reach, effectiveness, adoption, implementation, maintenance
SD: Standard deviation
SGRQ: St George’s respiratory questionnaire
6MWT: Six-minute walk test
